# Supplementary figures and images for: WHO Grade II or III Solitary Fibrous Tumors (Hemangiopericytomas) of the Spine: Two Case Reports with a Comprehensive Review of the Literature
Source: J Clin Med. 2025 Aug 27;14(17):6068. doi: 10.3390/jcm14176068 (PMC12429495; doi:10.3390/jcm14176068)

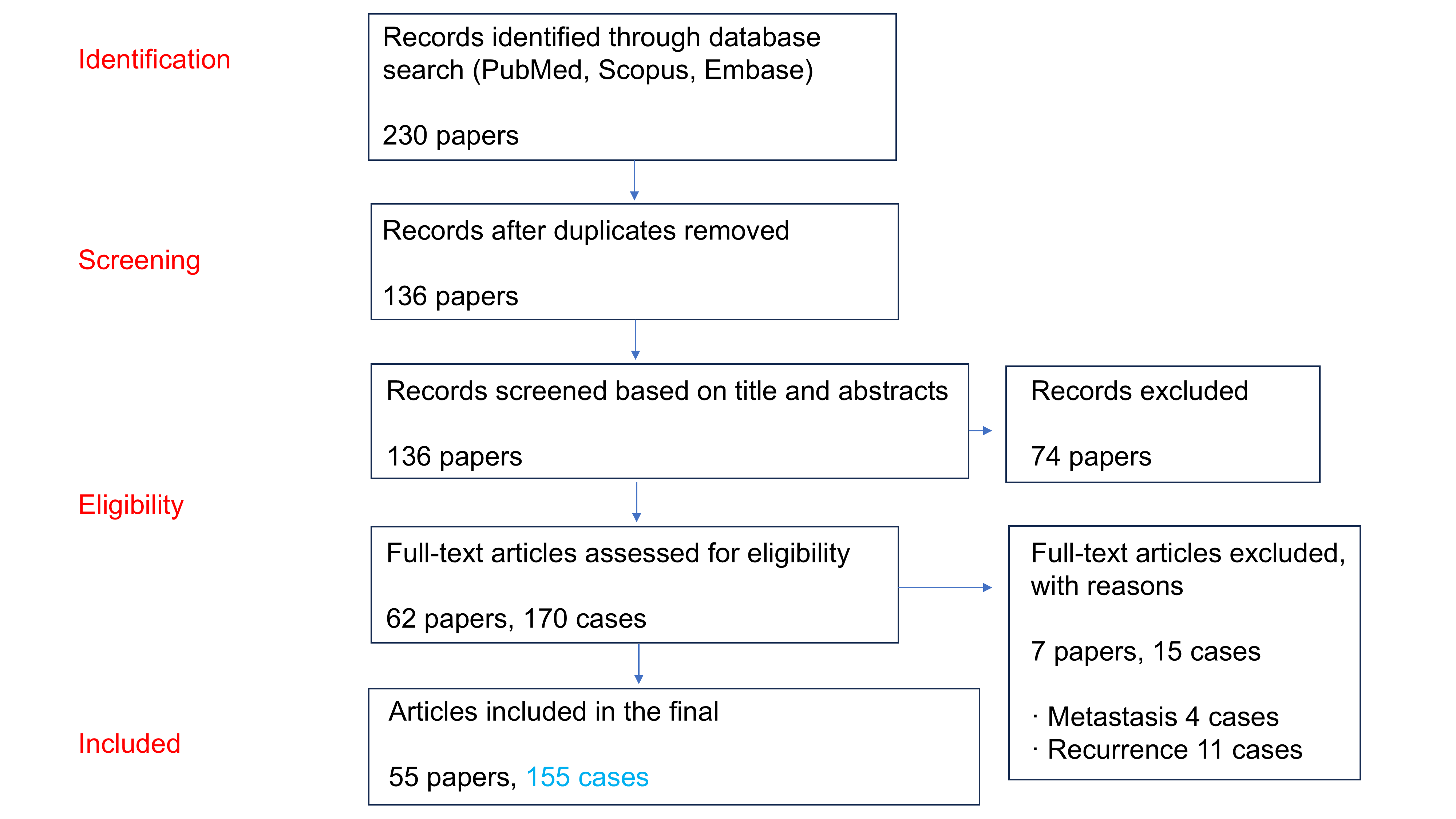

Supplement: Supplementary file 1 [file jcm-14-06068-s001.zip › jcm-3771600-supplementary.tif]
